# Glycogen synthase kinase-3β regulates fractalkine production by altering its trafficking from Golgi to plasma membrane: implications for Alzheimer’s disease

**DOI:** 10.1007/s00018-016-2408-6

**Published:** 2016-11-10

**Authors:** Almudena Fuster-Matanzo, Jerónimo Jurado-Arjona, Stefano Benvegnù, Esther García, Patricia Martín-Maestro, Raquel Gómez-Sintes, Félix Hernández, Jesús Ávila

**Affiliations:** 1 Present Address: Department of Clinical Neurosciences, NIHR Biomedical Research Centre, Wellcome Trust-Medical Research Council Stem Cell Institute, University of Cambridge, Clifford Allbutt Building, Cambridge Biosciences Campus, Cambridge, CB2 0PY UK; 2 Department of Molecular Neurobiology, Centro de Biología Molecular Severo Ochoa (CSIC-UAM), 28049 Madrid, Spain; 3 Centro de Investigación Biomédica en Red sobre Enfermedades Neurodegenerativas (CIBERNED, ISCIII), Madrid, Spain; 4 Departament of Cellular and Molecular Biology, Centro de Investigaciones Biológicas, CSIC, 28040 Madrid, Spain

**Keywords:** GSK-3β, Fractalkine, Golgi network, Rab8, Alzheimer’s disease

## Abstract

Glycogen synthase kinase-3β (GSK-3β) is a serine-threonine kinase implicated in multiple processes and signaling pathways. Its dysregulation is associated with different pathological conditions including Alzheimer’s disease (AD). Here we demonstrate how changes in GSK-3β activity and/or levels regulate the production and subsequent secretion of fractalkine, a chemokine involved in the immune response that has been linked to AD and to other different neurological disorders. Treatment of primary cultured neurons with GSK-3β inhibitors such as lithium and AR-A014418 decreased full-length fractalkine in total cell extracts. Opposite effects were observed after neuron transduction with a lentiviral vector overexpressing the kinase. Biotinylation assays showed that those changes mainly affect the plasma membrane-associated form of the protein, an observation that positively correlates with changes in the levels of its soluble form. These effects were confirmed in lithium-treated wild type (wt) mice and in GSK-3β transgenic animals, as well as in brain samples from AD patients, evident as age-dependent (animals) or Braak stage dependent changes (humans) in both the membrane-bound and the soluble forms of the protein. Further immunohistochemical analyses demonstrated how GSK-3β exerts these effects by affecting the trafficking of the chemokine from the Golgi to the plasma membrane, in different and opposite ways when the levels/activity of the kinase are increased or decreased. This work provides for the first time a mechanism linking GSK-3β and fractalkine both in vitro and in vivo, with important implications for neurological disorders and especially for AD, in which levels of this chemokine might be useful as a diagnostic tool.

**Electronic supplementary material:**

The online version of this article (doi:10.1007/s00018-016-2408-6) contains supplementary material, which is available to authorized users.

## Introduction

GSK-3 protein is a proline-directed serine/threonine protein kinase. In mammals there are two GSK-3 isoforms encoded by two different genes, GSK-3α with a molecular weight of 51 kDa and GSK-3β with a molecular weight of 47 kDa [1]. GSK-3 can phosphorylate a variety of cytoplasmic and nuclear proteins and its substrates include cytoskeletal proteins, transcription factors and metabolic regulators. Thus, GSK-3 plays important roles both in embryonic development and in adulthood [2]. Dysregulation of GSK-3 activity is believed to play a key role in the pathogenesis of chronic central nervous system disorders, such as bipolar disorder, Huntington’s disease and importantly in AD [3, 4]. Recently, different groups have documented that GSK3β activity is also crucial to regulate the inflammatory response by either promoting or inhibiting the process through the expression of pro- and anti-inflammatory cytokines, respectively [5]. However, no connection between the kinase and fractalkine, an important chemokine in the inflammatory response, has been established so far.

Fractalkine, also known as neurotactin or CX3CL1, is an exceptional member of the large family of chemokines, with high expression levels in the brain [6]. Different cell types have been reported to express the protein including neurons [7]. Fractalkine exists in both soluble and membrane-associated forms. The 95 kDa full-length protein is a type I transmembrane protein consisting of N-terminal chemokine domain, a glycosylated mucin-like stalk, a transmembrane region and an intracellular C-terminal domain. The soluble form is approximately 70 kDa and comprises the mucin-like stalk and the chemokine domain which can be released from the full-length protein via the action of several proteases, such as cathepsin S, ADAM10, and ADAM17 in both the periphery and the central nervous system [8–10]. Important functions have been described for both forms of the protein. While the membrane-anchored form acts as an adhesion molecule promoting retention of leucocytes to endothelial cells under physiological flow conditions [11], the soluble form exhibits chemotactic activity [12, 13]. Fractalkine binds to a single receptor subtype, CX3CR1, expressed by myeloid cells including microglia [14], and interestingly, both soluble and transmembrane forms of the protein have been shown to ligate CX3CR1 [9, 10]. In the brain, this interaction controls microglia activation in basal conditions. However, depending on the type of injury, the fractalkine/CX3CR1 axis plays a different role in neurodegeneration *versus* neuroprotection [15]. Indeed, contradictory results have been obtained in different mouse models of AD [16, 17]. Thus, further studies are required to elucidate the implication of this chemokine-receptor interaction in the disease. In the context of mood disorders, the potential utility of fractalkine as a pathologically relevant biomarker or therapeutic target has been recently proposed for future clinical and translational research [18].

Considering the different roles played by the transmembrane and the soluble forms of fractalkine in the inflammatory response, it is of great importance to unravel the mechanisms responsible for the balance between both pools, particularly those regulating the availability of the chemokine at the plasma membrane which may directly influence the levels of the secreted form. Here we have identified GSK-3β as a novel regulator of fractalkine production and subsequent secretion, whose decreased activity or increased levels mediate opposite outcomes in the amount of both transmembrane and soluble forms in neurons. In line with this, we show for the first time that Rab8^+^ vesicles are responsible for fractalkine transport from the *trans* Golgi to the plasma membrane, with GSK-3β being an active modulator of this transport. Interestingly, similar results regarding the levels of membrane-bound and soluble fractalkine were obtained in vivo with remarkable age dependent differences. Finally, a similar pattern was observed in brain samples from AD patients at different Braak stages. These findings reveal a novel and interesting GSK-3β-mediated regulatory pathway for fractalkine, a chemokine that might be explored as a useful candidate for the early detection of AD.

## Materials and methods

### Reagents

The following reagents were used: AR-A014418 (Sigma), lithium chloride (Sigma), MG132 (Calbiochem), bafilomycin A1 (Sigma), Akt inhibitor VIII (Millipore), EZ-link Sulfo-NHS-LC-Biotin (Pierce), Dynabeads^®^ M-280 Streptavidin (Thermo Fisher), complete Proteases Inhibitor (Roche), Lipofectamine 2000 (Invitrogen), DuoSet ELISA Kit for human samples and DuoSet ELISA Kit for mouse samples (R&D Systems).

### Immunofluorescence and confocal microscopy

Cells were fixed with 4% paraformaldehyde diluted in PBS at room temperature for 15 min. They were permeabilized/blocked with PBS/0.1% Triton X‐100/1% (w/v) bovine serum albumin (BSA) for 1 h. Subsequently, cells were incubated overnight at 4 °C with the following primary antibodies: anti-mouse Fractalkine 1/150 (R&D systems), anti GM-130 1/200 (Abcam), anti TGN38 1/200 (NovusBio) and Rab8 1/200 (Santa Cruz Biotechnologies). After three washes with 1× PBS, cells were incubated for 1 h at room temperature with Alexa Fluor-conjugated secondary antibodies (Invitrogen), washed three times with 1× PBS and incubated with DAPI (Merck) for 10 min. Stained cells were analyzed with a Zeiss LSM710 Vertical confocal microscope using a 63× objective and a 2.5 zoom. Single planes showing the greatest proportion of Golgi cisternae (either *cis* or *trans*) or Rab8^+^ vesicles were selected to acquire representative images.

### Animals

#### Animal care and treatments

Mice were bred at the Centro de Biología Molecular and treated following the guidelines of Council of Europe Convention ETS123. GSK3β mice were generated as described previously [19]. Mice were kept on a normal light–dark cycle (12 h light/12 h dark), with free access to food and water. Male and female mice were used equally in all the experiments.

Two-month-old wild type C57BL/6 mice were fed with chow containing 1.7 g LiCl/kg (Harlan Teklad) for 2 weeks, followed by a diet containing 2.55 g LiCl/kg for 6 weeks. Control mice were fed with lithium-free chow under parallel conditions. Blood lithium levels were analyzed weekly by inductively coupled plasma mass spectrometry (ICP-MS). To prevent hyponatremia, water and a NaCl solution (450 mM) were available ad libitum.

Three-month-old and 13-month-old GSK3β mice were used as representative groups of young and old animals, respectively.

### Human subjects

The use of human brain tissue samples in this study was coordinated by the local Brain Bank (Banco de Tejidos CIEN, Madrid), following national laws and international ethical and technical guidelines on the use of human samples for biomedical research purposes [20]. In all cases, brain tissue donation, processing and use for research was performed in compliance with published protocols [21].

### Western blot

Western blot analyses were prepared by homogenizing samples in ice-cold extraction buffer consisting of 50 mM Tris HCl, pH 7.4, 150 mM NaCl, 1% NP-40, 1 mM sodium orthovanadate, 1 mM EDTA, a protease inhibitor cocktail and 1 µM okadaic acid. Protein concentration was determined by a BCA Protein Assay kit (Thermo Scientific). 30 µg of total protein were electrophoresed on 10% SDS–polyacrylamide gel and transferred to a nitrocellulose membrane (Schleicher & Schuell, Keene, NH). The following primary antibodies were used: anti-mouse Fractalkine (R&D systems), anti-human Fractalkine (R&D systems), anti-PHF-1 (Tau P-Ser396/404) 1/100 (kind gift from Dr. Peter Davies, NY, USA), anti-Phospho-GSK-3α/β (Ser21/9) (1/500) (Cell signaling), anti GSK-3α/β (1/1000) (Cell signaling), anti-Phospho-Akt (Ser473) 1/500 (Cell Signaling), anti-Akt 1/1000 (Cell Signaling), anti-β tubulin (1/5000) (Sigma), anti-GAPDH 1/10,000 (Abcam) and anti-flotilin-1 1/500 antibody (Abcam). Membranes were incubated with the antibody at 4 °C overnight in 5% nonfat dried milk. Secondary goat anti-mouse, anti-rabbit (1/1000; Invitrogen, San Diego, CA) and secondary donkey anti-goat (1/1000 Santa Cruz Biotech) antibodies were used. ECL detection reagents (Amersham Biosciences, Arlington Heights, IL) were used for immunodetection. Quantification was performed with Image J software. Values were normalized with respect to the values obtained with an anti-β-tubulin antibody for total extracts and with an anti-flotilin 1 antibody for membrane pellets to correct for total protein content.

### Statistical analysis

A Kolmogorov–Smirnov test was performed for all data sets to test normality. When normal distribution was determined, comparisons between two independent samples were performed by a *t* test and comparisons among groups were done by performing a one-way ANOVA. Non-normal data sets were analyzed by equivalent nonparametric tests. *P* < 0.05 values were considered significant. The SPSS v.22 software (SPSS, 1989; Apache Software Foundation) was used for all statistical analyses.

Additional methods are detailed in Supplemental information section.

## Results

### GSK-3β regulates the amount of membrane-bound fractalkine with an impact on the levels of the soluble form

**Fig. 1:**
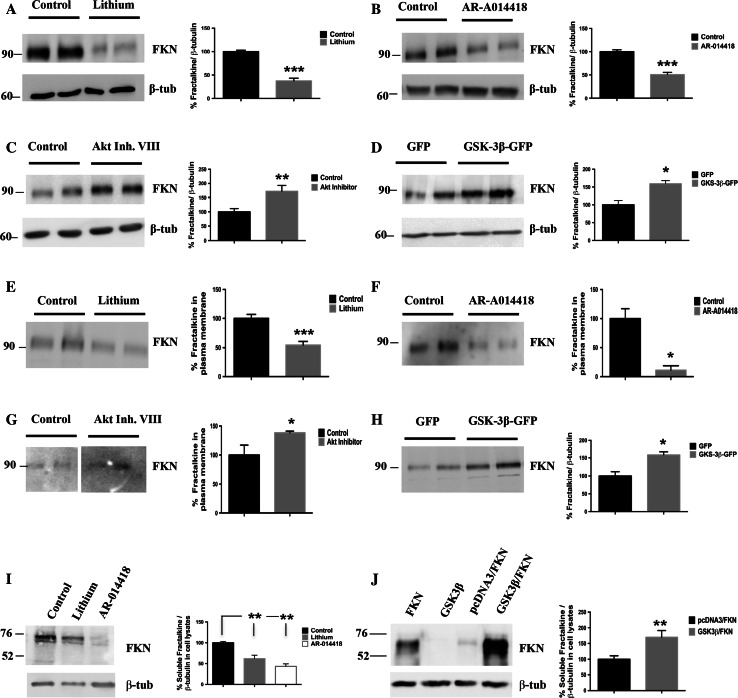
Effects of GSK-3β on soluble and transmembrane fractalkine levels in vitro. Inhibition with lithium (20 mM) (**a**) or AR-A014418 (33 μM) (**b**) decreases total levels of fractalkine in neuronal lysates. GSK-3β-increased activity by pharmacological approaches (Akt inhibitor VIII (5 μM)) (**c**) or genetic approaches (lentiviral transduction) (**d**) increases total fractalkine levels in neuronal lysates. Biotinylation assays show how GSK-3β-induced fractalkine changes mainly affect the membrane-bound form of the protein (**e**–**h**). Western blot detection of fractalkine in concentrated media derived from transfected HEK293 cells treated with lithium (20 mM) or AR-A014418 (33 μM) (**i**) or in co-transfection experiments (**j**) showing a correlation with the observed changes in the membrane-anchored form. FKN, Fractalkine; β-tub, β-tubulin; Li, lithium; AR, AR-A014418; Akt Inhib. VIII, Akt Inhibitor VIII. *P < 0.05; **P < 0.01; ***P < 0.001 *versus* control/GFP/pcDNA3-FKN samples. *N* ≥ 3 biological replicates. Data are expressed as mean ± SEM

Given the role of both GSK-3β and fractalkine in inflammatory processes and their implication in neurological disorders with a high inflammatory component, we wondered whether a possible link between the two proteins might exist. To investigate this, we first analyzed by Western blot, total extracts of primary cultured neurons treated with lithium, a classic general inhibitor of the kinase and with AR-A014418, a more specific inhibitor [22] (see Fig. S1). As it is shown in Fig. 1a, b, GSK-3β inhibition led to a dramatic decrease (around 50%) in the levels of the mature membrane-bound form of fractalkine according to its molecular weight (95 kDa). Opposite effects were detected when GSK-3β activity was increased by either a pharmacological approach through inhibition of Akt (a well-known upstream regulator of the kinase; Fig. 1c, Fig. S1) or by a genetic approach through neuron transduction with a GSK-3β overexpressing lentiviral construct (Fig. 1d, Fig. S1). In both cases, fractalkine levels were found increased. These results pointed out to a regulation of fractalkine production mediated by the kinase. To clarify if the affected fractalkine pool was the membrane-bound one, we performed biotinylation assays. As it is observed in Fig. 1e–h, both inhibition and activation of GSK-3β (via pharmacological approach or lentiviral transduction) resulted in the same effects previously observed in total extracts, confirming that the kinase indeed affects the amount of the transmembrane form. Since full-length fractalkine (95 kDa) is cleaved at the plasma membrane to give rise to a soluble secreted form (70 kDa) [8, 9], we wondered if GSK-3β induced changes in the membrane-bound pool could have an impact on the amount of soluble fractalkine. To address this, we analyzed media derived from fractalkine-expressing HEK293 cells treated with either lithium or AR-A014418, and from HEK293 cells co-expressing GSK-3β and fractalkine-expressing plasmids. Figure 1i, j shows how decreased fractalkine at the plasma membrane caused by GSK-3β inhibition is reflected in decreased levels of the soluble form, while increased levels of the membrane-bound form are translated into increased soluble fractalkine levels.

### GSK-3β regulates Golgi-mediated fractalkine trafficking

**Fig. 2:**
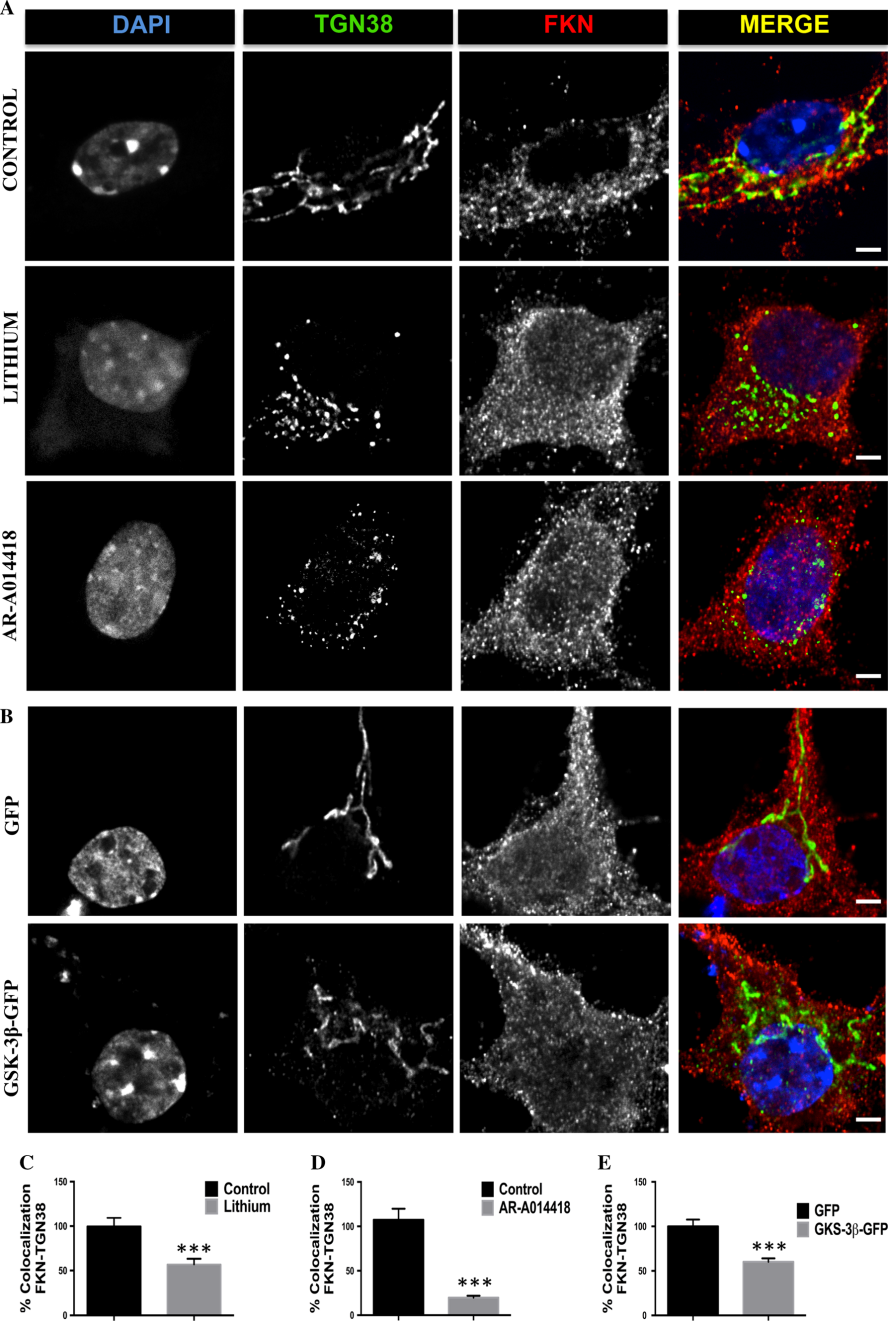
Effects of GSK-3β on fractalkine localization in the *trans* Golgi network. Immunostaining with *trans* Golgi marker TGN38 and fractalkine antibody showing an increased colocalization between both markers in neurons where the kinase was inhibited either by lithium (20 mM) or AR-014418 (33 μM) (**a**) and in neurons transduced with control GFP or GSK-3β-GFP lentiviruses (**b**). *Graphs* representing the results of the colocalization analysis in treated (**c**, **d**) or transduced neurons (**e**). *Right column* in **a** and **b** shows merge images. *P < 0.05; **P < 0.01 *versus* control. *Scale bar* 3 μm. *N* ≥ 3 biological replicates. Data are expressed as mean ± SEM

To obtain possible clues about the GSK-3β-mediated mechanisms regulating fractalkine production and considering that the main protein degradation pathways were not responsible for the observed changes in fractalkine levels (Fig. S2), we performed an immunofluorescence assay in primary cultured neurons upon kinase inhibition/overexpression. Interestingly, in some neurons lithium and AR-A014418 caused fractalkine signal to accumulate in the perinuclear region (Fig. S3A–C), a typical location for the Golgi apparatus, where indeed protein glycosylation and maturation processes normally take place [23]. Surprisingly, GSK-3β overexpression did not produce any remarkable change in fractalkine localization compared to GFP transduced control neurons (Fig. S3D, S3E). In an attempt to further characterize this possible Golgi-related phenomenon in GSK-3β inhibited neurons, we decided to perform colocalization experiments using typical markers of both *cis* and *trans* Golgi cisternae. The staining with the antibodies GM-130 and TGN-38 (*cis* and *trans* Golgi, respectively) revealed a fragmented pattern (Fig. S4A and Fig. 2a, respectively), something that had been previously described in HeLa cells [24]. However, in our model, this fragmentation preferentially affected the *trans* Golgi. Indeed, TGN38 staining revealed different fragmentation stages that in some cases, particularly corresponding to AR-A014418 treated neurons were more evident (Fig S5). We then quantified the percentage of positive GM-130 or TGN-38 vesicles that were also positive for fractalkine staining. Results regarding GM-130 did not reveal any difference between control and treated neurons (Fig. S4C, S4D). However, quantification with TGN-38 antibody resulted in a significant decreased colocalization in neurons treated with both inhibitors (Fig. 2c, d). In some cases, fractalkine staining gave the similar fragmented pattern observed with Golgi antibodies. In general, fragmentation was so severe that this fractalkine pattern was not observed anymore. These results confirmed that decreased GSK-3β activity was interrupting normal fractalkine processing due to Golgi disruption.

We next tried to find out if Golgi-related effects were also present in GSK-3β transduced neurons. To test that we stained GFP and GSK-3β-GFP transduced neurons with GM-130 and TGN-38 antibodies, and we analyzed the percentage of colocalization with fractalkine antibody. Images and graphs in Figs. S4B and 2B show that in this case, increased GSK-3β levels did not produce any noticeable difference in Golgi morphology, but again decreased the percentage of fractalkine colocalization with *trans* Golgi antibody (Fig. 2e) without affecting its colocalization with GM-130 (Fig. S4E). These results together with the increased levels found in total extracts and in biotinylation experiments, might be indicative of a putative accelerated fractalkine trafficking from the *trans* Golgi network to the plasma membrane.

**Fig. 3:**
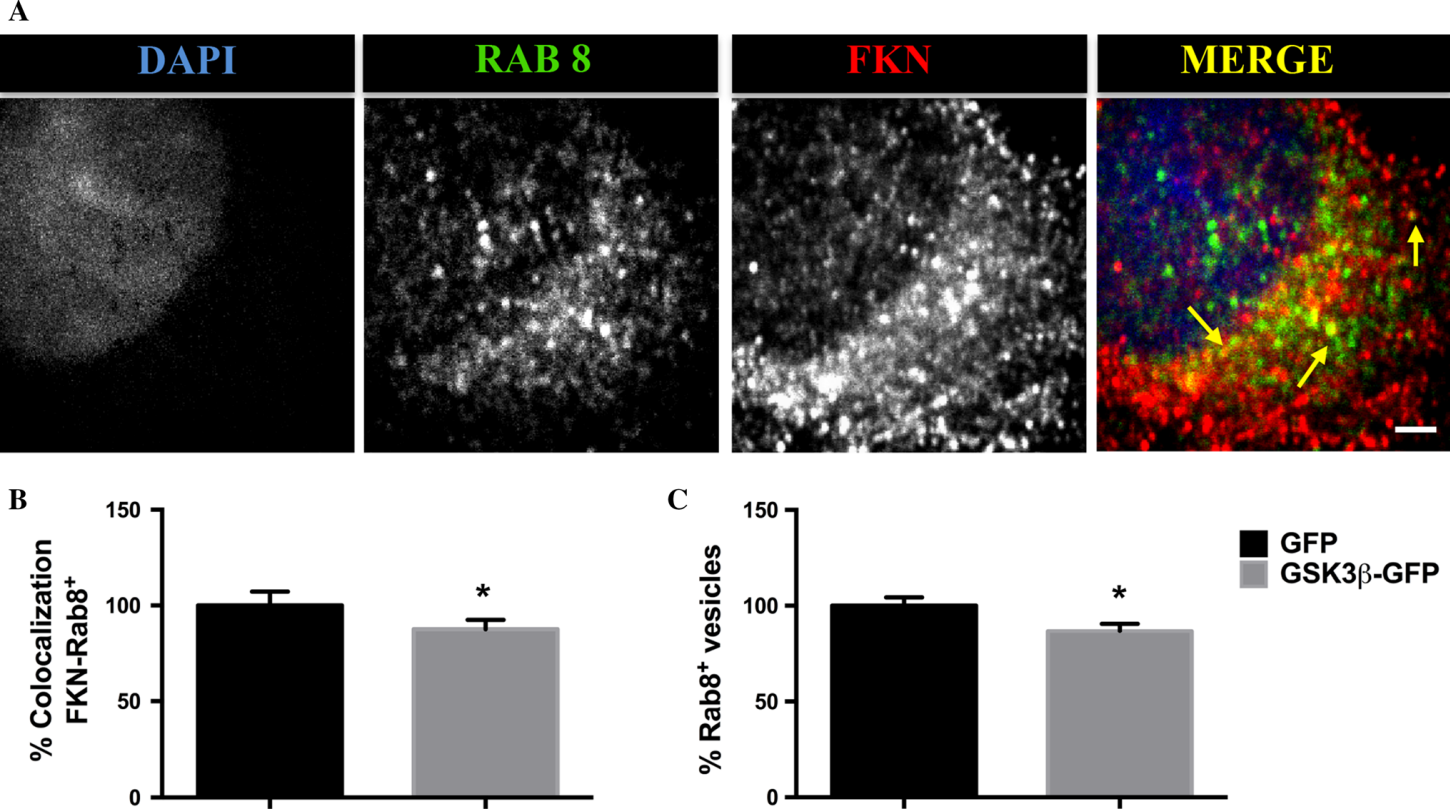
GSK-3β overexpression affects fractalkine colocalization in Rab8^+^ vesicles. Immunostaining with Rab8 and fractalkine antibodies in GSK-3β-GFP transduced neurons. * Arrows* show colocalization between both markers (**a**). GSK-3β decreases fractalkine colocalization with Rab8^+^ vesicles (**b**). Total Rab8^+^ vesicle pool is affected by GSK-3β upregulation (**c**). *P < 0.05 *versus* GFP transduced neurons. *Scale bar* 3 μm. *N* ≥ 3 biological replicates. Data are expressed as mean ± SEM

Rab GTPases are master regulators of the complex network of pathways that confirm the intracellular membrane traffic [25]. With the aim of testing the hypothesis of an accelerated fractalkine trafficking, we decided to pay attention to Rab8, a small GTPase that has been previously described to be involved in vesicular traffic between the *trans* Golgi cisternae and the plasma membrane [26]. As illustrated in Fig. 3a, a significant pool of Rab8^+^ vesicles colocalizes with fractalkine, confirming the implication of this GTPase in the transport of the protein, something that had not been described so far. We then performed another set of immunofluorescence assays, this time by analyzing the colocalization rates between Rab8 and fractalkine markers. Quantifications demonstrated that this percentage is lower in GSK-3β transduced neurons compared to the control (Fig. 3b). Furthermore, the total number of Rab8^+^ vesicles under GSK-3β overexpression was also decreased (Fig. 3c). Together these results support the idea of an accelerated fractalkine trafficking as a consequence of an upregulated GSK-3β activity.

### GSK-3β-mediated fractalkine changes are also observed in vivo

**Fig. 4:**
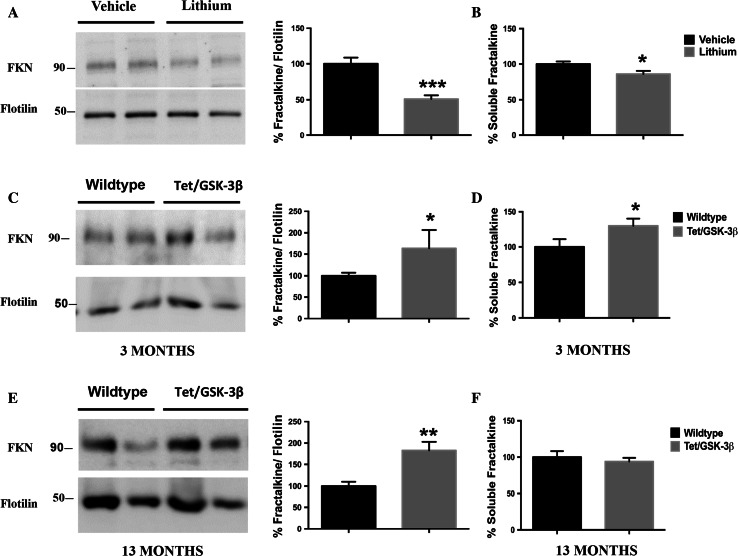
Effects of GSK-3β on soluble and transmembrane fractalkine levels in vivo. GSK-3β inhibition also decreases fractalkine levels in membrane pellet extracts from wt animals treated with lithium (**a**). Tet/GSK-3β transgenic animals show increased fractalkine levels in membrane pellet extracts from 3-month-old animals (**c**) and 13-month-old mice (**e**). *Graphs* showing quantification of soluble fractalkine measured by ELISA in soluble enriched brain samples from wt animals treated with lithium (**b**) or 3-month-old (**d**) and 13-month-old (**f**) Tet/GSK-3β animals. A correlation between the soluble fractalkine and the membrane-bound form levels was found in lithium-treated animals and young transgenic mice. *FKN* fractalkine. *P < 0.05; **P < 0.01; ***P < 0.001 *versus* control. Lithium-treated mice, *N* = 9 per group; 3-month-old transgenic animals, wt *N* = 13, Tet/GSK-3β *N* = 10; 13-month-old animals, *N* = 6 per group. Data are expressed as mean ± SEM

Since our in vitro experiments demonstrated clear effects on both fractalkine membrane-bound form and fractalkine soluble form in cultured neurons mediated by GSK-3β, we wondered if the same results would occur in mice. For this purpose, we first analyzed lithium-treated animals and collected membrane extracts and soluble enriched fractions from brain samples. Western blot analysis revealed that GSK-3β inhibition led to a ~50% decrease in fractalkine levels (Fig. 4a, Fig. S6), which correlated with a concomitant decrease in the soluble form (Fig. 4b), confirming our previous in vitro results.

We next studied the same issue in a model with GSK-3β overexpression in the forebrain (Tet/GSK-3β mice) that was previously demonstrated to display an Alzheimer’s disease-like phenotype [27]. Since the over-activation of the kinase has been reported to be a key factor in many neurodegenerative diseases in which some of the alterations show a temporal pattern [28], we decided to evaluate fractalkine levels in 3-month-old and 13-month-old mice. Interestingly, increased levels in membrane extracts were detected in both young (Fig. 4c) and old mice (Fig. 4e). However, only in 3-month-old mice, increased membrane levels positively correlated with increased soluble levels (Fig. 4d), while in old mice no differences in the soluble enriched fractions were detected (Fig. 4f).

These results demonstrate how GSK-3β activity regulates membrane-bound fractalkine in vivo and how this is translated into changes in the soluble form of the protein only in young animals.

### Alzheimer’s disease samples display similar fractalkine-related alterations

**Fig. 5:**
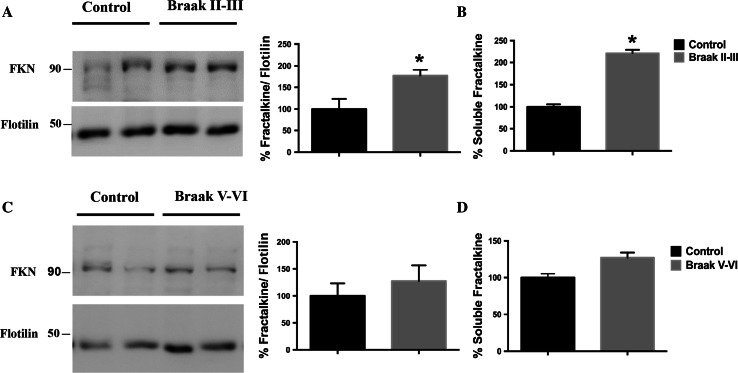
Alzheimer’s disease human samples also show altered transmembrane and soluble fractalkine levels. Fractalkine changes differ according to AD progression with increased levels in membrane pellet samples from II to III Braak stage (**a**) and no changes in V–VI Braak stage (**c**). *Graphs* representing the quantification of soluble fractalkine measured by ELISA in soluble enriched brain samples showing increased fractalkine levels in II–III Braak stage samples (**b**) and no changes in V–VI Braak stage Alzheimer’s disease patients (**d**). *FKN* fractalkine. *P < 0.05; **P < 0.01; ***P < 0.001 *versus* control. Total *N* = 19 (see Fig. S7). Data are expressed as mean ± SEM

Regarding the above-mentioned implication of GSK-3β in AD, as well as the controversial role of fractalkine signaling in the disease, and taking into account our in vitro and in vivo results, we finally studied fractalkine levels in brain extracts from patients of different Braak stages (see Fig. S7). Importantly, we found a similar pattern to the one observed in Tet/GSK-3β mice. Thus, in early Braak II–III stages, increased levels of both membrane-associated (Fig. 5a) and soluble fractalkine (Fig. 5b) levels were found. Interestingly, in the later stages no statistical differences were detected in either membrane-bound (Fig. 5c) or soluble protein (Fig. 5d).

Altogether these results confirm a regulation of fractalkine in AD brains that might be due to the dysregulated activity of GSK-3β in these patients. Furthermore, these results strongly support the link between soluble fractalkine levels and AD progression, pointing to GSK-3β as an important modulator of this phenomenon during the disease.

## Discussion

Several important roles have been attributed to fractalkine/CX3CR1 communication in both health and disease of the central nervous system, with most of the studies to date having paid special attention to the receptor. In this study, we demonstrate how the amount of both membrane-anchored and soluble fractalkine depends on GSK-3β activity/levels, a kinase which has been proposed as a key element in AD pathology [29] and with many other implications in other neurological disorders as well [28]. Our results indicate how GSK-3β regulates the availability of fractalkine at the plasma membrane, and as a result, the amount of the cleaved form in the medium, by interfering in the protein processing/maturation through the Golgi network.

The Golgi apparatus is a central intracellular membrane organelle for trafficking and modification of proteins and lipids which functions as a central hub in the exocytic secretory pathway [30]. Here, we have shown how modifications in GSK-3β activity/levels may impact in Golgi-mediated fractalkine trafficking in two different and opposite ways. First, in good agreement with the work of Adachi et al., our results demonstrate how Golgi morphology is largely affected by the decrease in GSK-3β activity which results in both *cis* and *trans* cisternae fragmentation [24]; although as our results demonstrate, this fragmentation preferentially affects the *trans* network in neurons, resulting also in decreased immunofluorescence colocalization with fractalkine antibody particularly affecting this portion of the structure. Specifically, regarding the TGN38 marker, we have been able to observe different fragmentation stages that may represent the sequential events followed by the neuron in response to GSK-3β inhibition. In the end, this phenomenon leads to a complete disaggregation and disappearance of the *trans* Golgi network, interrupting the normal exocytic pathway followed by fractalkine.

When the kinase levels and/or activity are increased, the opposite effects occur. While no changes were detected in either the morphology of the Golgi apparatus or in the colocalization assays with GM-130 marker, a decrease was observed with TGN38, pointing to specific effects related to the *trans* Golgi network. Considering that total fractalkine levels are increased, and specifically, levels corresponding to the membrane-bound form, we argue that GSK-3β overexpression might be affecting the sorting of the chemokine. Indeed, it is known that a portion of GSK-3β localizes in the *trans* Golgi, where it interacts with some Golgi associated proteins such as p230 [24] and MACF1 [31]. However, effects have been mainly described in knock down experiments. In the opposite scenario, the one that we describe in this work, increased GSK-3β levels and activity would raise the phosphorylation status of its putative substrates that in turn, might affect the functioning of the organelle. For example, the oligomerization of the Golgi reassembly stacking proteins (GRASP proteins) is regulated by phosphorylation [32]. Furthermore, several studies highlight their importance in Golgi structure maintenance and architecture which is required for accurate glycosylation and sorting [33]. Although no interaction between GRASPs and GSK-3 has been reported so far, they constitute a good example of how phosphorylation of Golgi associated proteins may control the functioning of the organelle.

Our work also highlights for the first time that the small GTPase Rab8 contributes to fractalkine transport from the *trans* Golgi to the plasma membrane. We have first demonstrated how fractalkine colocalizes with Rab8^+^ vesicles and how the total number of these vesicles is reduced when GSK-3β is overexpressed in neurons. Although further experiments are required to confirm a GSK-3β-induced accelerated fractalkine trafficking from the Golgi apparatus to the plasma membrane, our results greatly support this hypothesis. In this scenario, accelerated trafficking would lead to an increased fusion rate of the Rab8^+^ vesicles to the plasma membrane, causing a decrease in their total number as well as in the colocalization between them and fractalkine protein, and consequently, leading to higher levels of the fractalkine membrane-bound pool. Finally, supporting this idea it has been previously reported that GSK-3β favors the exchange of GDP/GTP for Rab8 [34], something that would enhance the activity of this latter and might contribute to the above-mentioned acceleration in this pathway.

Importantly, GSK-3β variations affect fractalkine levels also in animal models. This might be relevant on one hand, in the context of neuropsychiatric disorders, where lithium has been traditionally used as a therapeutic drug [35]. On the other hand, results in Tet/GSK-3β mice might be interesting for the early diagnosis of AD. In these animals, age-related variations in the levels of both the membrane-bound and the soluble form of fractalkine correlate with what it is observed in AD samples from early and late Braak stages. We argue that the discrepancy in the results concerning the membrane-bound form in the late Braak stages comparing to the transgenic model might be due to the multifactorial aspect of the pathology [36]. Curiously, both in old animals and in Braak V–VI human samples, we do not observe any difference in fractalkine soluble levels. This could be explained by the decreased activity of some of the metalloproteases mediating fractalkine cleavage. Indeed, ADAM-10 has been suggested as a valuable target for the prevention/treatment of AD, where mutations attenuating its activity and decreased levels have been described in the pathology [37]. Our results are also in good agreement with a paper published in 2008, where the level of soluble fractalkine in plasma was significantly greater in the patients with mild to moderate AD than in the patients with severe AD [38]. As a whole, all these results might be easily interpreted taking into account the neuroprotective role that has been extensively attributed to fractalkine [39]. Thus, in the earliest stages of the disease, elevated levels of the chemokine could constitute a protecting response against the initial injury. With time and pathology progression, these levels would decrease becoming undifferentiated from control patients in the later stages.

In conclusion, our results stress the importance of finely balanced GSK-3β levels for fractalkine delivery and how the increase or the decrease in its activity result in opposite effects with direct consequences in the total levels of both the membrane-bound and the soluble form. These effects are also present in vivo as well as in AD brain samples. As a consequence, fractalkine might not be considered as a simple neuroprotective responsive element but as an interesting diagnostic marker for AD instead.

### Electronic supplementary material

Below is the link to the electronic supplementary material.

Supplementary material 1 (TIFF 2226 kb)

Supplementary material 2 (TIFF 11108 kb)

Supplementary material 3 (TIFF 4601 kb)

Supplementary material 4 (TIFF 12314 kb)

Supplementary material 5 (TIFF 8201 kb)

Supplementary material 6 (TIFF 805 kb)

Supplementary material 7 (DOCX 34 kb)

## Abbreviations

AD: Alzheimer’s disease
Aβ: Amyloid β
APP: Amyloid precursor protein
Cdk-5: Cyclin-dependent-like kinase 5
GSK-3β: Glycogen synthase kinase-3β
GRASP: Golgi reassembly and stacking protein
