# Supplementary figures and images for: Glycogen synthase kinase-3β regulates fractalkine production by altering its trafficking from Golgi to plasma membrane: implications for Alzheimer’s disease
Source: Cell Mol Life Sci. 2016 Nov 10;74(6):1153–63. doi: 10.1007/s00018-016-2408-6 (PMC5309299; doi:10.1007/s00018-016-2408-6)

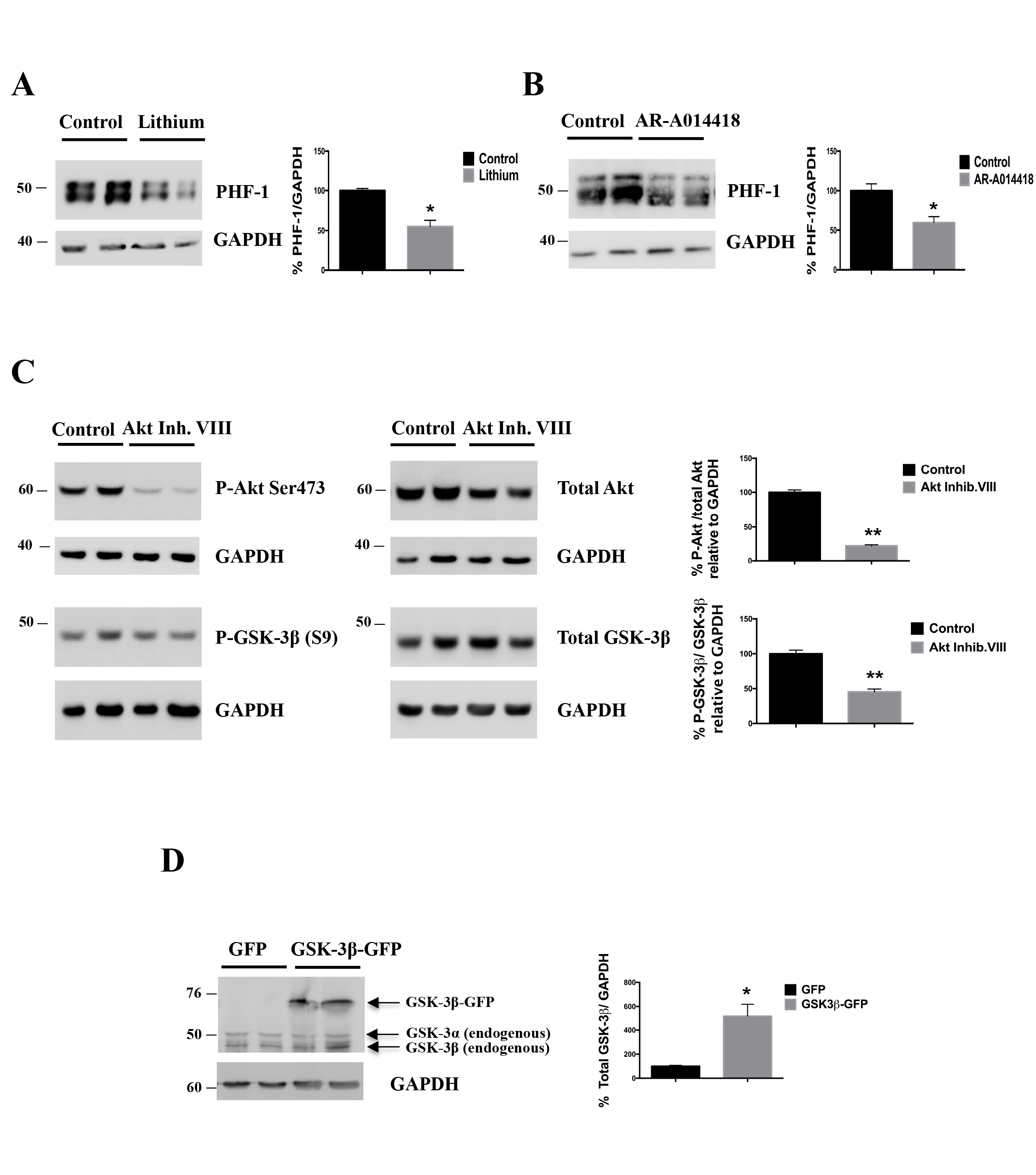

Supplement: Supplementary file 1 — Supplementary material 1 (TIFF 2226 kb) [file 18_2016_2408_MOESM1_ESM.tif]

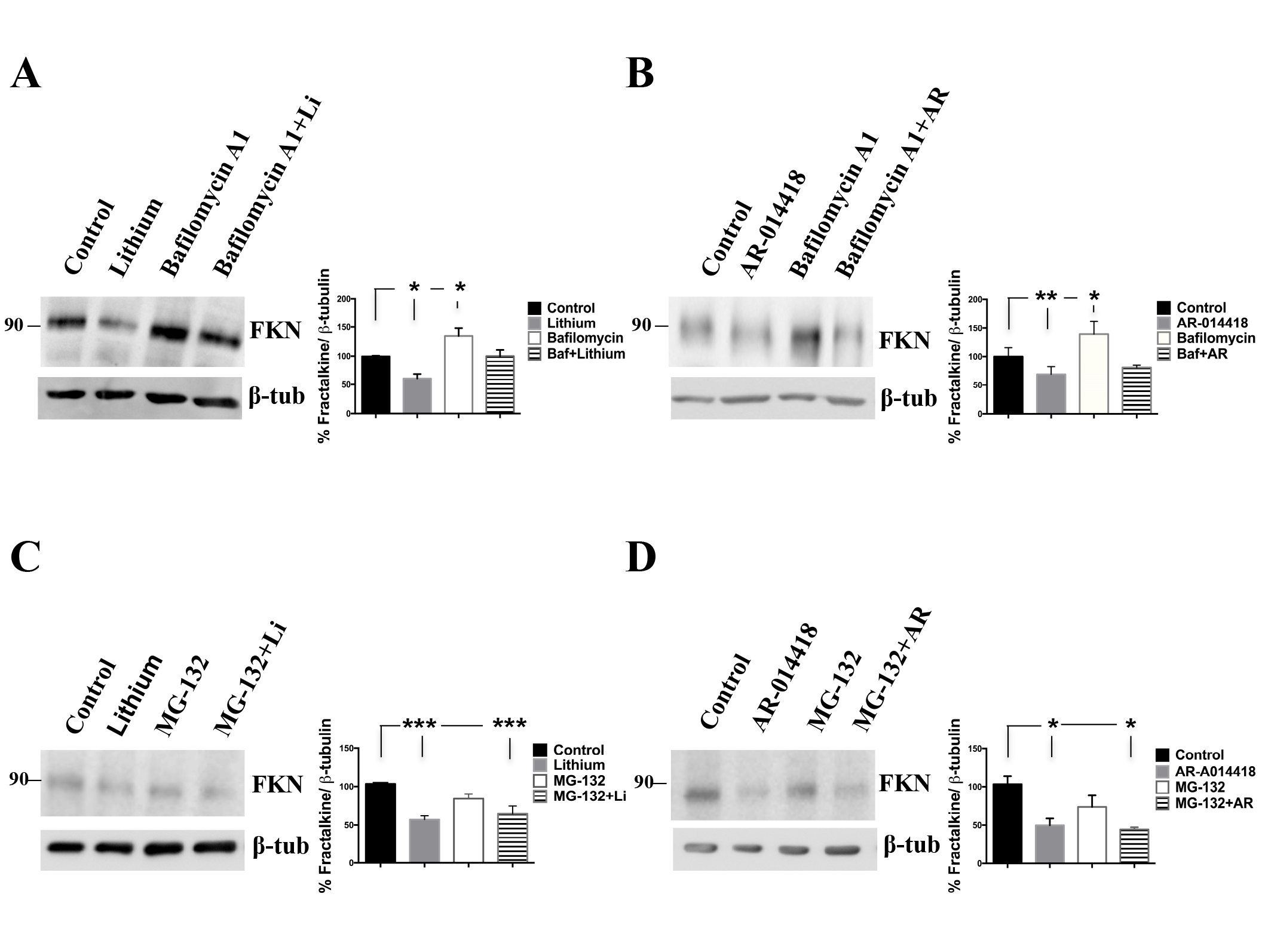

Supplement: Supplementary file 2 — Supplementary material 2 (TIFF 11108 kb) [file 18_2016_2408_MOESM2_ESM.tif]

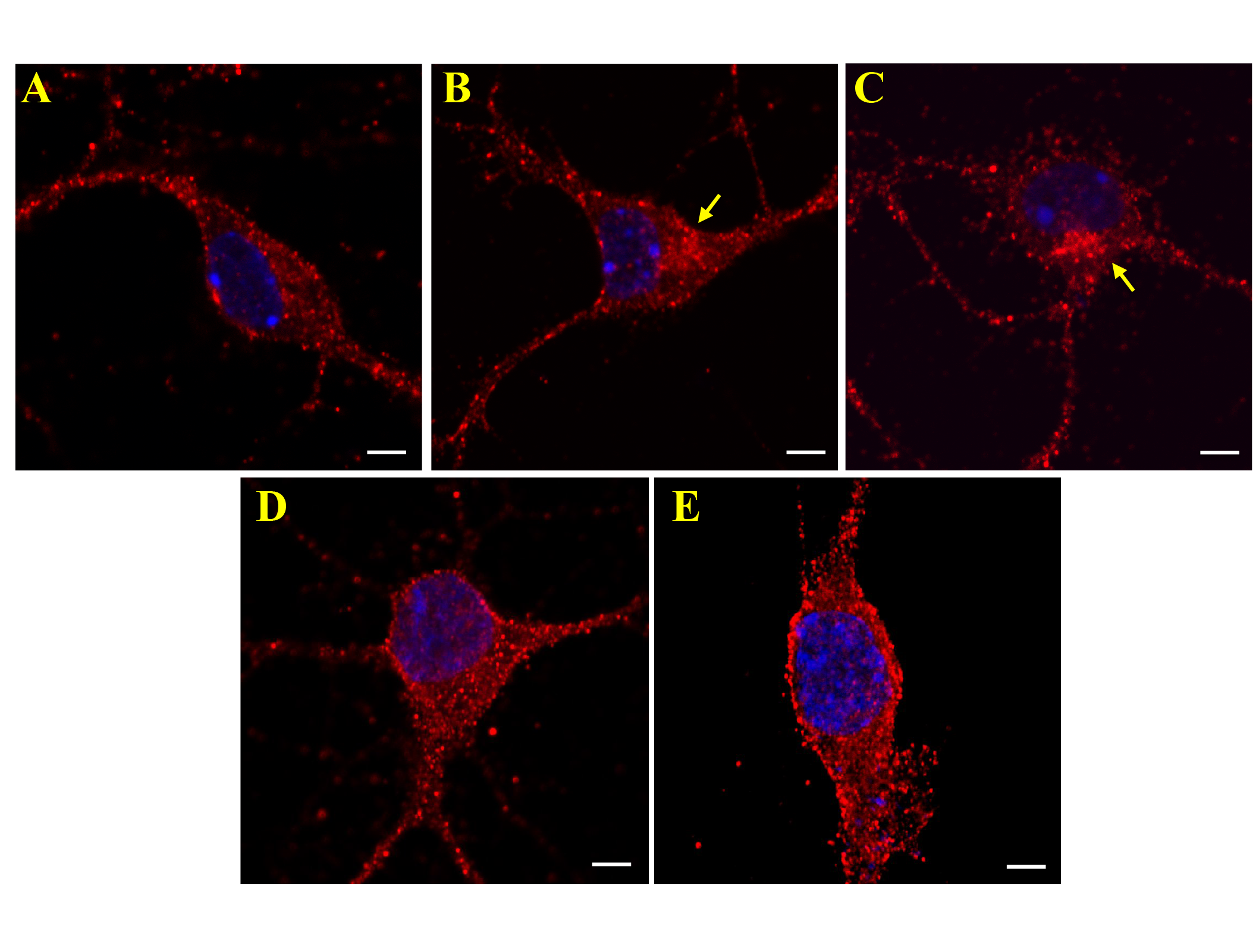

Supplement: Supplementary file 3 — Supplementary material 3 (TIFF 4601 kb) [file 18_2016_2408_MOESM3_ESM.tif]

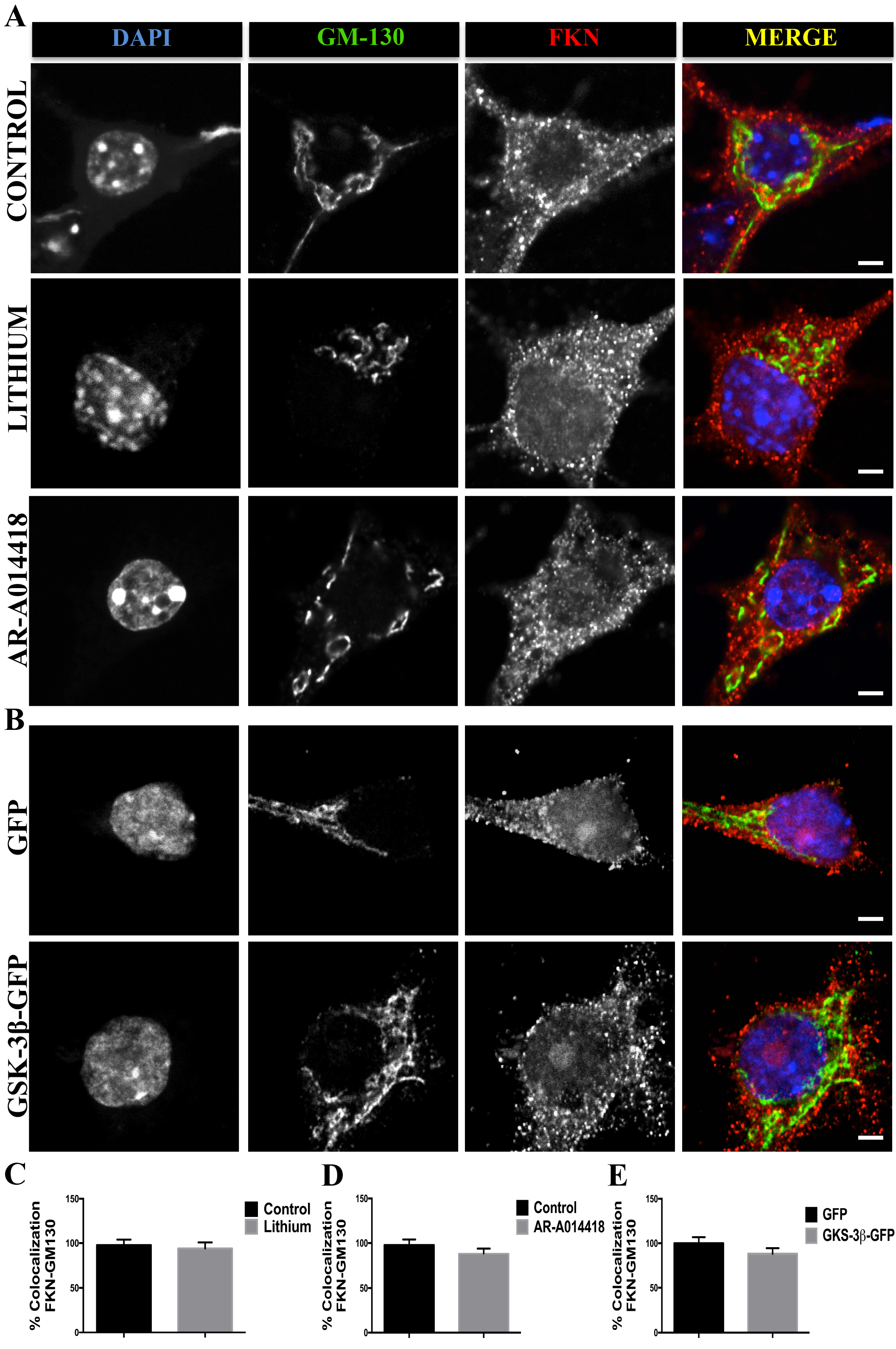

Supplement: Supplementary file 4 — Supplementary material 4 (TIFF 12314 kb) [file 18_2016_2408_MOESM4_ESM.tif]

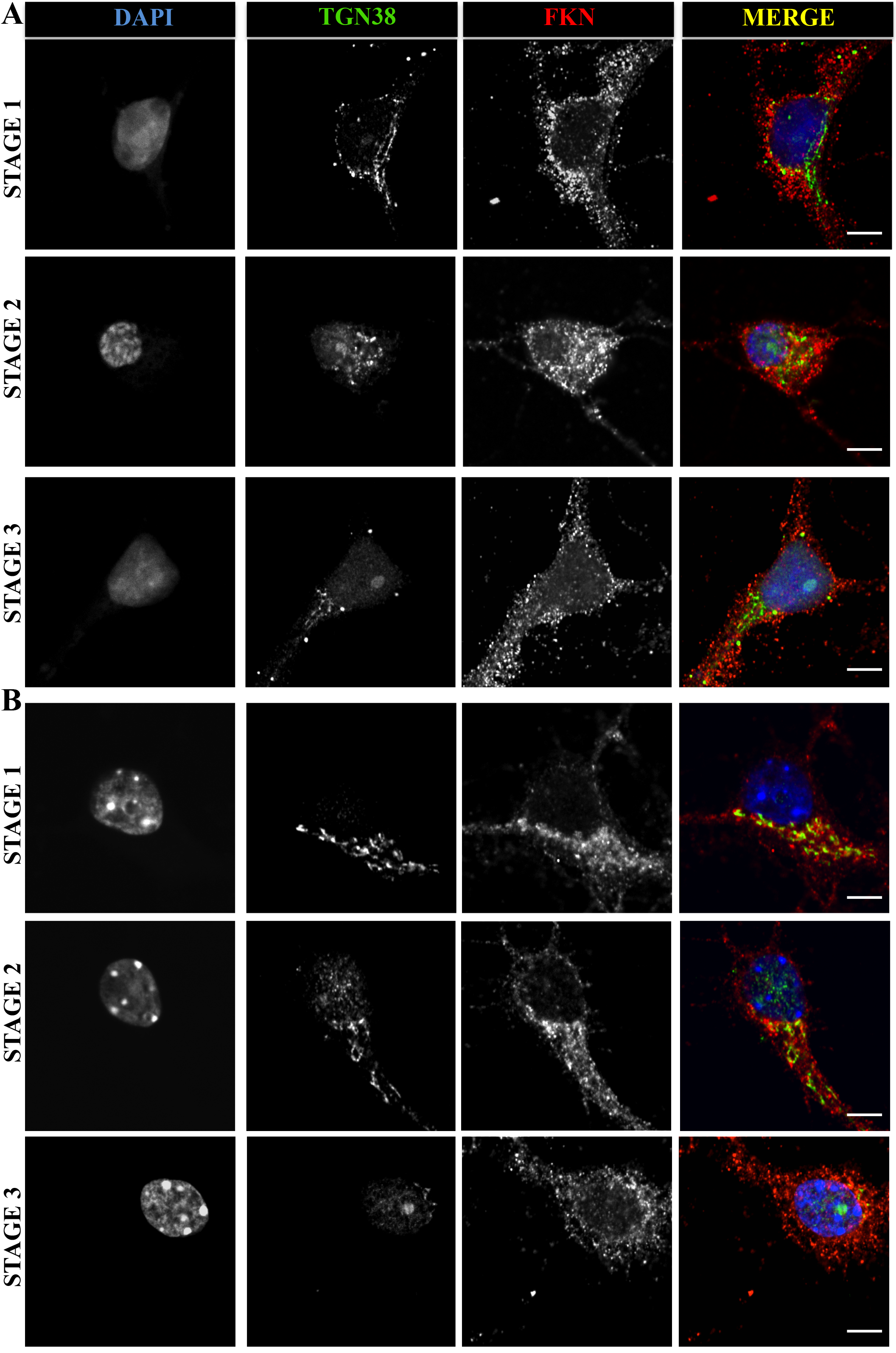

Supplement: Supplementary file 5 — Supplementary material 5 (TIFF 8201 kb) [file 18_2016_2408_MOESM5_ESM.tif]

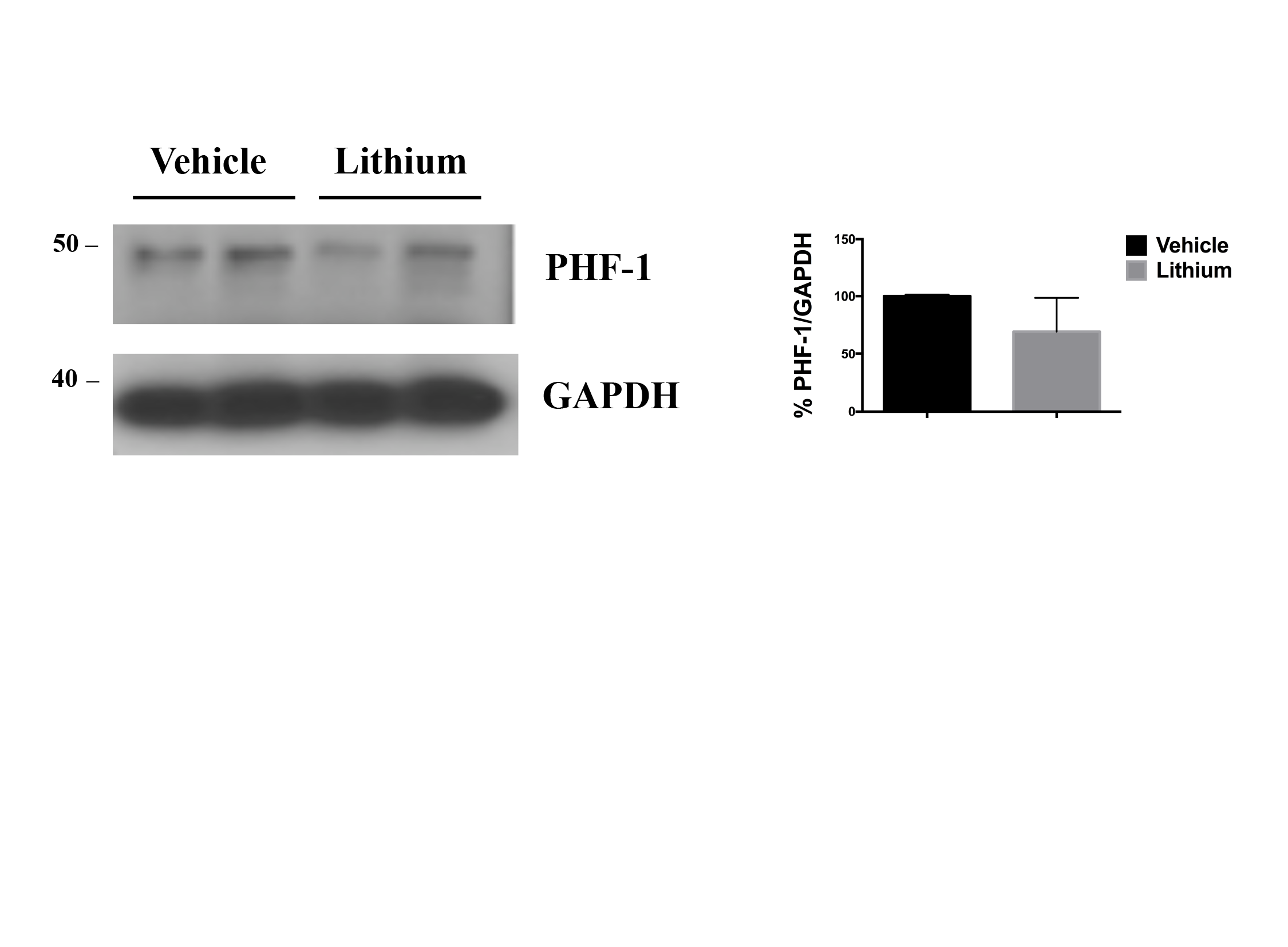

Supplement: Supplementary file 6 — Supplementary material 6 (TIFF 805 kb) [file 18_2016_2408_MOESM6_ESM.tif]
