# Supplementary material for: Glycogen synthase kinase-3β regulates fractalkine production by altering its trafficking from Golgi to plasma membrane: implications for Alzheimer’s disease
Source: Cell Mol Life Sci. 2016 Nov 10;74(6):1153–63. doi: 10.1007/s00018-016-2408-6 (PMC5309299; doi:10.1007/s00018-016-2408-6)
Supplement: Supplementary file 7 — Supplementary material 7 (DOCX 34 kb) [file 18_2016_2408_MOESM7_ESM.docx]

**SUPPLEMENTAL INFORMATION**

**SUPPLEMENTAL EXPERIMENTAL PROCEDURES**

**Plasmids, transfection and transduction**

Fractalkine plasmid was a kindly gift provided by Prof. Andreas Ludwig (Aachen, Germany). pcDNA3.1 was acquired from Invitrogen (Invitrogen, Carlsbad, CA, USA). pcDNA3/GSK-3β was previously described [1]. HEK293 cells were transfected with Lipofectamine 2000 following the manufacturer’s protocol. GSK-3β lentiviral plasmid was purchased from Origene. Lentiviral particles were produced according to the manufacturer’s instructions. Neurons were transduced at 4 DIV and medium was fully changed with neuronal conditioned medium after infection

**Cell culture**

HEK293 *Homo sapiens* embryonic kidney cells and ﬁbroblast-like (ATCC, Rockville, MD, USA) were routinely grown in Dulbecco’s modiﬁed Eagle’s medium, containing 10% fetal bovineserum (FBS), 2 mM of L-glutamine, 100 U/mL penicillin, and100 mg/mL streptomycin at 37°C in a 5% CO_2_ atmosphere. Exceptionally, for medium concentration experiments, after transfection cells were maintained in Dulbecco’s modiﬁed Eagle’s medium without FBS. Primary cultures of neurons were prepared according to modified versions of established procedures [2]. Briefly, embryonic brain tissue of E18 wt was collected and both hippocampus and cortices were extracted and dissociated individually from each embryo with the papain dissociation system (Worthington Biochemical Corp.; Lakewood, NJ). Neurons were maintained in Neurobasal medium (Invitrogen) supplemented with 1% B-27, 5% FBS, 0.5 mM glutamine, 100 units/ml penicillin, and 100 mg/ml streptomycin, and they were grown on 10 μg/ml poly-L-lysine-coated plates. The cells were incubated in 95% air, 5% CO2 in a humidified incubator at 37 °C. Cytosine arabinoside (2.5 μM) was added to the cultures on the third day after seeding (DIV3) to inhibit the proliferation of non-neuronal cells, and the cells were used for experiments at DIV11. Treatments with the different inhibitors were performed as follows: Lithium (5h); AR-014418 (20h); Akt Inhibitor VIII (8h); Bafilomycin (6h alone; 1h of pretreatment with Bafilomycin + 5h with Lithium; 14h of pretreatment with AR-014418 + 5h with Bafilomycin), MG-132 (17 h alone; 12h of pretreatment with MG-132 + 5h with Lithium; 3h of pretreatment with AR-014418+ 17h with MG-132).

**Biotinylation assay**

Plates were transfer to ice, medium removed and were washed three times with ice cold 1X PBS pH 7.9. They were then incubated with 0.5 mg/ml biotin for twenty minutes on ice on a vertical shaker in the cold room, washed again three times and incubated with ice cold 20 mM glycin prepared in 0.1% BSA 1X PBS. After two additional washes neurons were lysed with STEN-Lysis Buffer + Protease inhibitors, incubated for 10 min on ice and centrifuged 15 min at 13200 rpm at 4°C. For the immunoprecipitation, protein concentration was determined by a BCA Protein Assay kit (Thermo Scientific) and 200 ug were added equally in all cases to 20 ul of streptavidin beads. Immunoprecipitation was performed overnight at 4°C on an overhead shaker. Next, samples were washed twice with STEN NaCl and one time with STEN buffer. Beads were resuspended in 10 ul of loading dye and loaded on a 8% SDS-polyacrylamide gel.

**Medium concentration**

1 ml of medium collected from the experiments of single- and double-transfections in HEK293 were centrifuged at maximum speed for 5 min and protease inhibitors were added. Supernatant was then transferred to a Centricon filter (Sartorius) and centrifuged at 12630 rpm for 10 min following manufacturer’s instructions. Recovered medium was mixed with loading dye and the same volume of medium was loaded on a 8% SDS-polyacrylamide gel.

Three biological replicates were measured. Each biological sample was measured in triplicate.

**Colocalization assay**

Colocalization analysis was performed with ImageJ software (Bethesda, MD, USA), and every cell was manually delimited according to fractalkine staining. The background of different channels was edited with the Subtract Background tool with a rolling ball radius established for every independent culture; using a threshold intensity, binary images were obtained. The logical operation AND of the Image Calculator tool was used to generate an image harbouring only overlapping structures of both channels. Colocalization measurement was obtained by quantifying the area occupied by the overlapping elements per cell. At least 100 cells were measured for every condition and independent primary culture. Three biological replicates were analyzed.

**Tissue processing**

Animals were completely anaesthetized with an intraperitoneal penthobarbital injection (EutaLender, 60 mg/kg bw) and transcardially perfused with saline. Brains were removed and hippocampus were quickly dissected on an ice cold plate and frozen in dry ice. ~~Animals were killed and the cortices were quickly dissected on an ice-cold plate~~. Frozen human hippocampal samples were proportioned by the Brain Bank as indicated previously. Tissue fractioning protocol to obtain membrane pellets and an enriched soluble fraction was adapted from Lesné S *et al*. [3]. Briefly, hippocampal samples from either mice or humans were homogenized in a buffer containing 50mM Tris HCl pH 7.5, 150 mM NaCl, 2 mM EDTA, 1% NP-40, Tx-100 1% + protease inhibitors using a 1ml syringe (BD^TM^ Plastipak) and a 25g x 5/8" Needle (BD^TM^). Homogenates were centrifuge at 1000g for 10 min at 4°C. Supernatants were subjected to subsequent ultracentrifugation at 50000 g for 90 min at 4°C. Membrane pellets were collected after this process. Protein concentration was estimated in both soluble enriched and membrane pellet fractions using a BCA Protein Assay kit (Thermo Scientific). Pellets were resuspended in 50 ul of loading dye. Supernatants corresponding to soluble enriched fractions were used for ELISA assays.

**Enzyme-Linked Immuno-Sorbant Assay (ELISA)**

The CX3CL1 ELISA was performed using the DuoSet ELISA Kit (R&D Systems) according to the manufacturer’s instructions. All following steps were performed at room temperature. Enriched soluble fractions, standards and antibody were diluted in 2% BSA/PBS unless stated in the text. In brief, 2-5 μg/ml of capture antibody was coated to a Nunc MaxiSorbTM flat-bottom 96-well plate overnight in PBS. The plate was blocked in 2% BSA/PBS for 1-2 h before the samples and corresponding controls in different dilutions were incubated on the plate also for 1-2 h. Plates were washed three times with PBS. Following this, 200-400 ng/ml of detection antibody was added to the plate for another 1-2 h. Before the streptavidin-HRP was applied for 30 min, the plates were washed again three times with PBS. Substrate solution was added to the plate for 5-15 min and the reaction was stopped with the same volumes of 2N H_2_SO_4_. The optical density was measured in a GENios microplate reader at 450 nm and corrected by subtraction of the optical density at 550 nm. Data were normalized by the protein concentration determined in the soluble enriched fractions by BCA assay (see Tissue processing section ~~in Experimental Procedures~~).

**SUPPLEMENTAL REFERENCES**

[1] S Sanchez, CL Sayas, F Lim, J Diaz-Nido, J Avila, F Wandosell (2001) J Neurochem 78: 468.

[2] F Hernandez, M Perez, JJ Lucas, AM Mata, R Bhat, J Avila (2004) J Biol Chem 279: 3801. Doi:10.1074/jbc.M311512200

[3] S Lesne, MT Koh, L Kotilinek, et al. (2006) Nature 440: 352. Doi:10.1038/nature04533

**SUPPLEMENTAL FIGURES AND LEGENDS**

**Fig. S1.** Effectiveness of GSK-3β inhibitors and lentiviral constructs. GSK-3β was successfully inhibited with both Lithium (20mM) (A) and AR-014418 (33μM) (B) as demonstrated by the decreased phosphorylation levels in the PHF-1 site (Tau P-Ser396/404) of its main effector protein, tau. Akt Inhibitor VIII (5μM) decreases total phosphorylation levels in Serine 473 indicating a reduced activity of akt protein and a concomitant decreased phosphorylation in the inactivating Serine 9 residue of GSK-3β (C). Lentiviral transduction with GSK-3β-GFP construct leads to an increase in total GSK-3β levels compared to GFP-transduced control neurons (D). Li, lithium; AR, AR-A014418; Akt Inhib. VIII, Akt Inhibitor VIII. **P<*0.05; ***P<*0.01; *versus* control/GFP samples. N ≥ 3 biological replicates. Data are expressed as mean ± SEM.

**Fig S2.** GSK-3β mediated changes in fractalkine are not due to impairments in the main protein degradation systems. GSK-3β does not alter fractalkine lysosomal degradation as no changes in the levels of the cytokine were detected in neurons that were given a combined treatment ~~demonstrated in total neuronal extracts where neurons were treated with~~ of a lysosome inhibitor such as bafilomycin (200μM) ~~in combination~~ with either Lithium (20mM) (A) or AR-014418 (33μM) (B) ~~where fractalkine levels were not affected~~. Similar results were found for the proteasome degradation system in neurons treated with a proteasome inhibitor such as MG-132 (10μM) in combination with either Lithium (20mM) (C) or AR-014418 (33μM) (D). FKN, fractalkine; β-tub; β-tubulin; Baf, bafilomycin A1; Li, lithium; AR, AR-014418; **P<*0.05; ***P<*0.01 *versus* control. N ≥ 3 biological replicates. Data are expressed as mean ± SEM.

**Fig. S3.** GSK-3β variations cause differential effects in fractalkine ~~(in red)~~ distribution pattern. Immunofluorescence staining showing fractalkine (red) perinuclear localization (blue staining=DAPI) in neurons treated with lithium (20mM) (B) or with AR-014418 (33μM) (C) versus control neurons (A). No changes were found in neurons transduced with GFP (D) or GSK-3β-GFP lentiviruses (E). Arrows indicate fractalkine accumulations. Scale bar = 4 μm

**Fig. S4.** Effects of GSK-3β on fractalkine localization in the *cis* Golgi network. Fractalkine localization at *cis* Golgi cisternae remains invariable upon changes in GSK-3β protein levels/activity as it is demonstrated by the immunostaining with *cis* Golgi marker GM-130 and fractalkine antibody in neurons treated with either lithium (20mM) or AR-014418 (33μM) (A) and in neurons transduced with control GFP or GSK-3β-GFP lentiviruses (B). Graphs showing results of colocalization analysis in treated (C, D) or transduced neurons (E). Right column in A and B shows merge images. **P<*0.05; ***P<*0.01 *versus* control/GFP. Scale bar = 3 μm. N ≥ 3 biological replicates. Data are expressed as mean ± SEM.

**Fig. S5.** GSK-3β inhibition leads to Golgi apparatus fragmentation. ~~Image showing Golgi fragmentation pattern after GSK-3β inhibition with~~ Fractalkine (red), TGN38 (green) and DAPI (blue) immunostaining in neurons treated with either lithium (20mM) (A) ~~and with~~ or AR-014418 (33μM) (B) and the different fragmentation stages observed for each treatment. *Trans* Golgi appears differently fragmented from partial disintegration (stage 1) to almost completely disappearance of the organelle structure (stage 3) which is even more accurate with AR-014418 treatment (B). Scale bar = 5 μm

**Fig. S6.** Effectiveness of Lithium treatment on wt animals. GSK-3β was inhibited with Lithium (see Material and Methods section) as demonstrated by the decreased phosphorylation levels in the PHF-1 site of its main effector protein, tau. Total hippocampal extracts were analyzed for this purpose. N=5 per group; Data are expressed as mean ± SEM

**Fig. S7.** Table displaying the information related to human samples. Sex, Age, Condition (Control or Alzheimer) and Braak stage are shown in the table. ND= not defined.

| **Cod UIPA** | **Sex** | **Age** | **Condition** | **Braak stage** |
| --- | --- | --- | --- | --- |
| BCPA 65  BCM 81  BCM 15  BCPA0177  BCPA0206  BCPA0158  BCPA0025  BCPA0019  BCPA0014  BCPA0008  BCPA0018  BCPA0041  BCPA0202  BCPA0184  BCPA0030  BCPA0073  BCPA0139  BCPA0037  BCPA0151 | \| M \| \| --- \| \| M \| \| F \| \| F \| \| F \| \| M \| \| M \| \| F \| \| M \| \| M \| \| F \| \| F \| \| M \| \| F \| \| M \| \| F \| \| F \| \| M \| \| F \| | \| 41 \| \| --- \| \| 14 \| \| 49 \| \| 58 \| \| ND \| \| 84 \| \| 85 \| \| 85 \| \| 80 \| \| 87 \| \| 87 \| \| 73 \| \| 87 \| \| 81 \| \| 68 \| \| 88 \| \| 82 \| \| 77 \| \| 86 \| | Control  Control  Control  Control  Alzheimer  Alzheimer  Alzheimer  Alzheimer  Alzheimer  Alzheimer  Alzheimer  Alzheimer  Alzheimer  Alzheimer  Alzheimer  Alzheimer  Alzheimer  Alzheimer  Alzheimer | \| 0 \| \| --- \| \| 0 \| \| 1 \| \| 0 \| \| 3 \| \| 2 \| \| 3 \| \| 2 \| \| 2 \| \| 2 \| \| 5 \| \| 5 \| \| 5 \| \| 6 \| \| 6 \| \| 5 \| \| 5 \| \| 6 \| \| 6 \| |
